# Pediatric Suicide Attempt Non-Disclosure: an Analysis of Discrepant Screening Results

**DOI:** 10.1007/s11121-025-01817-8

**Published:** 2025-06-09

**Authors:** Anna Maria Ros, Rachel Ballard, Amanda Burnside, Michael Harries, Aron Janssen

**Affiliations:** 1 Northwestern Feinberg School of Medicine, Chicago, IL USA; 2 Ann & Robert H Lurie Children’s Hospital, Chicago, IL USA

**Keywords:** Universal screening, Suicide screening

## Abstract

The Ask-Suicide Screening Questions (ASQ) is a validated tool developed to assess suicidal risk in pediatric medical settings with one item assessing historical attempts. While the psychometric properties of the ASQ are well-established, little is known about how youth respond to this question upon repeated administrations. We conducted a retrospective analysis of electronic medical record data by youth who received the ASQ from December 2019 to November 2023 at an urban academic children’s hospital. Youth who disclosed a suicide attempt but denied an attempt history at a subsequent visit were identified. Multivariate regression and manual chart review were utilized to identify demographic and clinical variables related to non-disclosure of a previously disclosed attempt. Of 1861 encounters (1460 unique patients) with a disclosed historic suicide attempt, re-screening occurred in 503 future encounters. One hundred forty instances of nondisclosure occurred (127 unique patients). Encounters were classified into false positives (*N* = 26), encounters where nondisclosure by patients did not impact clinical response (*N* = 40), and encounters where nondisclosure resulted in no further suicide risk assessment (*N* = 74). Of this last group, 47.3% received no risk assessment at the initial visit. Compared to the initial visit, the nondisclosure visit was more likely to have a medical presenting complaint and to have negative responses on ASQ questions related to recent suicidal ideation. Denial of a historic attempt upon repeat administration of the ASQ is not uncommon among pediatric patients, and this is more likely to occur at an encounter for a medical presenting complaint.

## Introduction

Youth suicide is a leading cause of mortality, as the second leading cause of death for youth ages 10–14 and the third leading cause of death for youth ages 15–24 in the USA (CDC, 2023). American adolescents’ self-reported suicidal ideation, plans, and attempts are staggeringly high, as are suicide-related emergency room visits and inpatient admissions (Bommersbach et al., 2023; Gaylor et al., 2023). Relatedly, death by suicide in young people ages 10–24 increased by 56% between 2007 and 2017 (Curtin & Heron, 2019). Suicide attempts and death by suicide are common, yet preventable. The Joint Commission’s Sentinel Event Alert 56 recommends universal suicide risk screening in hospitals using brief, validated tools (2016). Previous literature has shown that universal screening for suicidality in the pediatric ED and general inpatient settings is feasible (Horowitz et al., 2020; Inman et al., 2019), and that positive suicide screens are associated with future suicide-related ED visits or death by suicide (DeVylder et al., 2019; Horowitz et al., 2022).

The Ask Suicide-Screening Questions (ASQ) measure is a validated, four item brief screening measure developed to assess suicidal ideation and behavior in pediatric medical settings. The ASQ has been validated across inpatient and outpatient settings and has a sensitivity of 96.9%, specificity of 87.6%, and negative predictive value of 99.7% (Horowitz et al., 2012). The ASQ includes three questions about suicidal thoughts in the past few weeks and one question about the patient’s lifetime history of suicidal behavior (Question #4: “Have you ever tried to kill yourself?”). In the setting of universal screening, an adolescent who visits the same ED on two subsequent days will be administered this screening measure at each novel ED encounter. In the outpatient setting, a patient who screens positive once will be re-screened at every subsequent visit. This means that for youth identifying a lifetime attempt, the NIMH clinical pathway suggests screening them at each visit, and there is little guidance currently on how often to administer these tools to the same patients. When the ASQ is administered to the same individual on multiple instances over time, a lack of consistency in response to this historical behavior question is clinically concerning, and little is known regarding how to interpret conflicting reports of lifetime behavior. Specifically, when an individual has a history of responding “yes” to question four, and subsequently answers “no,” this may indicate a limitation in the test–retest reliability of the item. It is additionally possible that aspects of the clinical response to the initial positive screen, or aspects of the administration of the following negative screen, have a dampening effect on the individual’s willingness to re-disclose previously described suicidal behavior.

The most potent predictor of future suicide is a previous attempt (Bostwick et al., 2016), which demonstrates the relevance and importance of assessing past attempts when screening for risk. Previous studies examining the psychometric properties of the ASQ have found that positive screens based on item four only may not accurately capture current severity, possibly due to the lifetime parameter of that question (Aguinaldo et al., 2021). Prior studies have investigated other discrepancies in screening, including denial of historic suicidal ideation in the period in which a suicide attempt occurred (Uhl et al., 2023), but there is a dearth of literature on within-person changes in reporting of historic previous suicide attempts upon repeated screenings. This is particularly true for youth in pediatric medical settings that have implemented universal suicide screening. In this retrospective analysis of emergency department and inpatient administrations of the ASQ, we initially set out to examine the frequency of previously disclosed suicidal behavior (changing from positive to negative responses on ASQ question four). We then set out to explore clinical correlations of non-disclosure; we hypothesized that visit type (medical or psychosocial), setting, and fidelity of clinical response to the initial disclosure might be related to episodes of nondisclosure of historic attempts. The results from our preliminary analyses led us to conduct a retrospective chart review to investigate the context of both disclosure and nondisclosure visits to further elucidate the phenomenon of subsequent nondisclosure of a previously disclosed suicide attempt in the context of universal suicide screening in a pediatric hospital. This manuscript highlights themes that were found across disclosure and nondisclosure visit pairs in our sample and discusses the clinical implications of nondisclosure of historic attempts in the setting of universal suicide screening.

## Methods

This study was conducted at a large, urban pediatric hospital where universal suicide risk screening is conducted using the ASQ in the Emergency Department (ED) and on inpatient medical floors for all youth 10 years of age and older in accordance with The Joint Commission’s Sentinel Event Alert 56 (2016). The ASQ is administered to youth upon admission to the ED as well as upon admission to the medical floor by nurses and medical assistants. If a patient is admitted to the hospital from the ED, they are re-screened when admitted to the medical floor regardless of whether the screening was positive or negative. Otherwise, screenings are not repeated during a single admission, unless indicated by clinical judgement. Screens are completed with youth independently from the caregiver and providers utilize a script consistent with NIMH recommendations to introduce the screening process. Following all positive screenings, a brief suicide safety assessment (BSSA) or full psychiatric consult is completed to characterize risk and determine next steps, which may include safety planning, referral to care, and safety precautions. In the absence of a positive ASQ screener, a BSSA will only be completed in cases where a clinician judges a patient to be at risk; a prior positive answer on question four will not result in further screening.

A positive response on the ASQ is defined as a “yes” or “refused to answer” code on any of the five questions, as specified by the toolkit (Horowitz, 2012). For youth who screen positive on any of the four items, an additional fifth item is administered to assess for imminent risk. A negative response on the ASQ screener is defined as a “no” code on all four questions. To examine instances of nondisclosure, all instances of a positive response on ASQ question four between January 2020 and October 2023 were identified and checked against sequentially subsequent administrations of the ASQ in either setting. Encounter pairs in which a positive response on question four was followed by a negative response on question four were included; the first visit was termed the disclosure visit and the second visit the nondisclosure visit.

We first extracted ASQ data from these visit pairs from the electronic medical record (EMR) together with demographic variables examined in other settings (age, race and ethnicity, insurance status, and binary gender as documented in the EMR; Roaten et al., 2021) and variables we hypothesized might be related to nondisclosure including setting of screening (emergency department vs inpatient medical units), medical versus psychosocial presenting complaints, and responses to the remaining items on the ASQ (current suicidal ideation). As positive suicide risk screening is known to be likelier with psychosocial than with medical presenting complaints (Roaten et al., 2021), we reviewed and categorized presenting complaints or admitting diagnoses recorded for each visit. Categories included medical, psychiatric, and social complaints and were independently reviewed by 2 physicians (RB and MH); differences in category assignment were resolved through discussion. Social complaints included terms such as “visit for assessment of child abuse,” “family discord,” and “school problems.” Psychiatric and social presenting complaints were then combined, resulting in a dichotomous variable indicating a medical versus psychosocial presenting complaint.

Investigators reviewed these data to examine associations with nondisclosure and determined that a retrospective chart review focused on records from the disclosure and nondisclosure visit, as well as any records available in the interval between these encounters, could help clarify context for these incidents of nondisclosure. Through an iterative process, our data analysis team, comprised of two clinical psychologists (AR and AB) and a triple board-certified child psychiatrist and pediatrician (RB), created a coding sheet to categorize characteristics of the disclosure and non-disclosure visits by first reviewing a set of 45 randomly selected visit pairs. We identified themes of interest including: (1) fidelity of clinical response to the initial disclosure (whether a clinical risk assessment was documented after the initial positive screening, either in the form of a BSSA or by a provider in the visit narrative), (2) who conducted the clinical risk assessment (a medical physician or a mental health provider), (3) whether the assessment included detail regarding the initial report of a history of suicidal behavior, and (4) disposition and recommendations for mental health follow-up care. Data was missing for disposition and recommendations for mental health follow-up in 38 encounters. Three categories of nondisclosure emerged through our retrospective chart review. In the first category, the initial disclosure appeared to have been a false positive due to an administrative error or the youth misunderstanding the question, or equating planning and preparatory activity with a suicide attempt. The second category involved nondisclosure visits in which patients either presented with chronic mental health concerns or other risk factors prompting a psychiatric evaluation despite nondisclosure. The third category we termed clinically significant nondisclosure, and this included all youth who did not receive an evaluation at the nondisclosure visit, but do not have documentation demonstrating the initial response was a false positive.

The study was reviewed and deemed exempt by the Institutional Review Board of Ann & Robert H. Lurie Children’s Hospital of Chicago (IRB 2021–4428).

## Statistical Analyses

Analyses included calculations of proportions of visits associated with any positive ASQ screen, with a positive response on ASQ question four, and in each of the three categories of nondisclosure. Multivariate logistic regression was used to identify the contribution of demographic variables and category of presenting complaints (medical, psychosocial) to all visits and those in each screening category. Odds ratios were calculated to highlight the strength of associations between demographic factors and ASQ positivity, question four positivity, and categories of nondisclosure outcomes. Analysis of the categories of nondisclosure (false positive initial disclosure, evaluated at nondisclosure visit due to known psychiatric history, and clinically significant nondisclosure) data coded from manual chart review of paired disclosure and nondisclosure visits included McNemar chi-square tests. Data analysis was conducted using Stata (StataCorp, 2023).

## Results

**Fig. 1:**
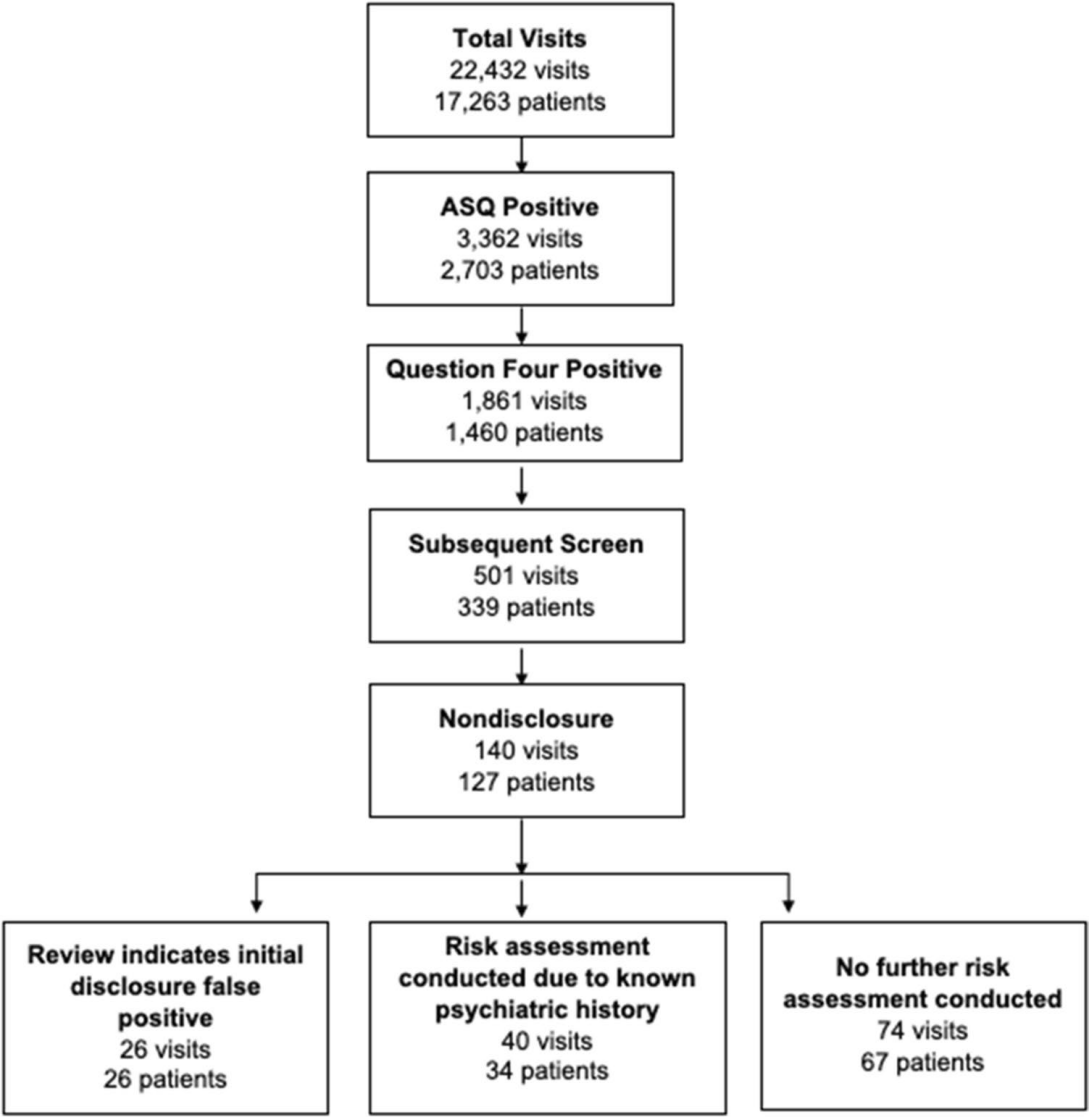
Breakdown of nondisclosure encounter categories

During the study period December 2019–October 2023, 23,432 ASQs were administered to 17,263 individual patients. Of these, 3362 (14.9%) ASQs resulted in a positive screening. There were 1861 (8%) encounters from 1460 unique patients with a positive response on question four, indicating a prior suicide attempt. Of these patients, 339 were subsequently re-screened in a total of 501 encounters. Of these encounters, there were 140 instances (28.7%) in which a patient gave a negative response to ASQ question four (nondisclosure encounter) and 357 instances (71.3%) in which a patient gave a positive response on question four consistent with their previous screen. Nondisclosure encounters (*N* = 140) were by 127 unique patients which included 6 patients with 2 nondisclosure visits, 2 patients with 3 nondisclosure visits, and 1 patient with 4 nondisclosure visits after their initial encounter. See Fig. 1 for a flowsheet of these encounters.

Demographic characteristics of all patients included in the study analysis are shown in Table 1. The likelihood for any positive screen as well as for a positive response on question four was greater among inpatients than ED patients (OR for any screen 1.65, *p* < 0.001, OR for question four 1.68, *p* < 0.001), and among Black, Non-Hispanic (OR for any screen 1.49, *p* < 0.001, OR for question four 1.46, *p* < 0.001), White, Non-Hispanic (OR for any screen 1.2, *p* < 0.001, OR for question four 1.42, *p* < 0.001) and Asian (OR for any screen 1.46, *p* < 0.05, OR for question four 1.57, *p* < 0.001) youth than for Hispanic youth. The odds ratio for any positive screen was also higher among patients with commercial insurance than with Medicaid (OR 1.16, *p* < 0.05), and positive screens were also more common among older age groups. The odds ratio for any positive screen was significantly higher for females than for males (OR, 0.45, *p* < 0.001) as was the odds ratio for a positive response on question four (OR 0.43, *p* < 0.001), and for clinically significant nondisclosure (OR 0.42, *p* < 0.001). The presenting complaint in visits with any positive screen and with a positive response on question four was significantly more likely to be psychosocial rather than medical (OR for any positive screen 34, *p* < 0.001, OR for positive response on question four 11.8, *p* < 0.001).

**Table 1:** Demographic characteristics and presenting complaints

	Full sample	ASQ positive		Q4 positive	
	*N* (%)	*N* (%)	OR (95% CI)	*N* (%)	OR (95% CI)
Visits	22,432	3362		1861	
Unique patients	17,263	2703		1460	
Setting					
ED	16,082 (71.7)	2513 (74.8)	Ref	1291 (69.4)	Ref
Inpatient	6350 (28.3)	849 (25.25)	1.69 (1.5–1.87)**	570 (30.6)	1.88 (1.66–2.12)**
Sex					
Female	12,040 (53.7)	2288 (68.1)	Ref	1340 (72)	Ref
Male	10,392 (46.3)	1074 (31.9)	.42 (.42–.51)**	521 (28)	.46 (.41-.52)**
Age					
7–12 years	4997 (22.3)	617 (18.3)	.68 (.6–.78)**	246 (13.2)	.47 (.4-.56)**
13–14 years	5307 (23.7)	848 (25.2)	.9 (.79–1.01)	439 (23.6)	.74 (.65-.85)**
15–16 years	6135 (27.4)	1038 (30.9)	Ref	640 (34.4)	Ref
17 + years	5993 (26.7)	859 (25.6)	.91 (.8–1.03)	536 (28.8)	.92 (.81–1.06)
Race/Ethnicity					
Hispanic	11,334 (50.5)	1286 (38.3)	Ref	688 (37)	Ref
Black, non-Hispanic	5320 (23.7)	1109 (33)	1.52 (1.34–1.74)**	585 (31.4)	1.48 (1.29–1.72)**
White, non-Hispanic	3680 (16.4)	586 (17.4)	1.25 (1.1–1.43)*	365 (19.6)	1.46 (1.26–1.69)**
Asian	795 (3.5)	130 (3.9)	1.5 (1.13–1.86)*	79 (4.3)	1.61 (1.22–2.12)*
Other	1303 (5.8)	251 (7.5)	1.32 (1.09–1.6)*	144 (7.7)	1.43 (1.16–1.77)*
Insurance Type					
Medicaid	14,077 (62.8)	1702 (50.7)	Ref	999 (53.7)	Ref
Commercial	8048 (35.9)	1613 (48)	1.15 (1.03–1.3)*	831 (44.7)	.94 (.84–1.07)
Self-pay/other	285 (1.3)	45 (1.3)	.71 (.47–1.07)	30 (1.6)	1.0 (.65–1.54)
Presenting Complaint					
Medical	19,249 (85.8)	1247 (37.1)	.03 (.03–.03)**	830 (44.6)	.08 (.07–.09)**
Psychosocial	3183 (14.2)	2115 (62.9)	ref	1031 (55.4)	ref

*p <.05

**p <.001

Chart review of all visit pairs including the initial visit (disclosure visit) and subsequent nondisclosure visit yielded three categories of clinical outcomes. We found that most commonly, pairs fell into the category of clinically significant nondisclosure (*N* = 74 encounters). Second most common (*N* = 40 encounters) were visit pairs in which the patient received a full risk assessment at the second (nondisclosure) visit despite the ASQ results. These visit pairs often represented patients with a psychiatric history known to the ED or inpatient staff, or where other risk factors prompted a full assessment (including other positive responses on the ASQ). False positives were least common (*N* = 26 encounters).

Demographic characteristics of patients in the three categories of nondisclosure visits are shown in Table 2. Demographic characteristics of each of the three nondisclosure visit categories were compared to the full sample of visits (*N* = 22,432) using the same logistic regression model used for Table 1. Visits with false positive disclosures were more likely to involve male patients (OR 1.82, *p* < 0.05). Visit pairs in which psychiatric assessment occurred regardless of negative screening at the nondisclosure visit were less likely to be patients in the youngest (OR 0.39, *p* < 0.05) and oldest (OR 0.47, *p* < 0.05) age categories, fewer with commercial insurance (OR 0.58, *p* < 0.05) and with more patients of Black, Non-Hispanic (OR 3.44, *p* < 0.001), White, Non-Hispanic (OR 2.79, *p* < 0.001), and Other race/ethnicity (OR 5, *p* < 0.001) backgrounds. Visit pairs in which nondisclosure of previously endorsed suicidal behavior resulted in no assessment were significantly less likely to be associated with male sex (OR 0.4, *p* < 0.001) and with the youngest age group (OR 0.22, *p* < 0.001).

**Table 2:** Demographics of patients with disclosure/nondisclosure visit pairs

	False positive disclosure		Evaluated at nondisclosure visit due to known psychiatric history		Clinically significant nondisclosure	
	*N* (%)	OR (95% CI) †	*N* (%)	OR (95% CI) †	*N* (%)	OR (95% CI) †
Nondisclosure visits	26 (100)		40 (100)		74 (100)	
Unique patients	26		34		67	
Sex						
Female	11 (42.3)	Ref	24 (60)	Ref	56 (75.7)	Ref
Male	15 (57.7)	1.82 (1.04–3.17)*	16 (40)	.93 (.59–1.47)	18 (24.3)	.4 (.27–.59)***
Age						
7–12 years	2 (7.7)	.52 (.22–1.29)	4 (10)	.39 (.18–.83)*	6 (8.1)	.22 (.11–.44)***
13–14 years	6 (23.1)	.93 (.45–1.93	12 (30)	1 (.49–1.68)	20 (27)	.75 (.49–1.15)
15–16 years	8 (30.8)	Ref	16 (40)	Ref	27 (36.5)	Ref
17 + years	10 (38.5)	1.07 (.53–2.15)	8 (20)	.47 (.24–.9)*	21 (28.4)	.88 (.6–1.3)
Race/ethnicity						
Hispanic	11 (42.3)	Ref	9 (22.5)	Ref	38 (51.4)	Ref
Black, non-Hispanic	7 (26.9)	.93 (.42–2.01)	14 (35)	3.44 (1.76–6.72)***	16 (21.6)	.96 (.59–1.57)
White, non-Hispanic	5 (19.2)	1.18 (.55–2.51)	10 (25)	2.79 (1.46–5.36)**	16 (21.6	1.2 (.79–1.83)
Asian	2 (7.7)	2.23 (.75–(6.66)	1 (2.5)	1.67 (.38–7.3)	2 (2.7)	.86 (.31–2.37)
Other	1 (3.9)	.61 (.14–2.64)	6 (15)	5 (2.37–10.68)***	2 (2.7)	.46 (.17–1.27)
Insurance type						
Medicaid	13 (50)	Ref	24 (60)	Ref	52 (70.3)	Ref
Commercial	13 (50)	1.24 (.66–2.34)	16 (40)	.58 (.35–.98)*	22 (29.7)	.73 (.48–1.1)
Other/self-pay	0(0)		0(0)		0(0)	

*CI* confidence interval, *OR* odds ratio

†Odds ratio as compared to the full sample shown in Table 1

*p <.05

**p <.01

***p <.001

Results from the chart review of visit pairs are shown in Table 3. Among visit pairs in which disclosure of a previous suicide attempt on ASQ question four was determined to be a false positive, all 26 patients were evaluated by a psychiatric consultant. The presenting complaint for the disclosure visit was medical for 7 (26.9%) visits and psychiatric for 19 (73.1%) visits, and 2 (6.5%) of the disclosure visits included imminent risk (ASQ question five positive). Documented examples of false positive responses on ASQ question four included: the patient having experienced suicidal ideation with planning and/or preparation but not an attempt; the patient having accessed lethal means but not used them; or simply further assessment indicating no history of past suicide attempts despite the positive response on question four. Chart review did not indicate that intellectual disability or developmental disorders were a factor in false positive responses on question four. All but one of the patients in this group received recommendations for follow-up psychiatric care after the initial visit.

**Table 3:** Characteristics of disclosure/nondisclosure visit pairs

	False positive disclosure	Evaluated at nondisclosure visit due to known psychiatric history	Clinically significant nondisclosure
Nondisclosure visits	26 (100)	40 (100)	74 (100)
Patients with nondisclosure visits	26	34	67
Setting			
Disclosure visit (*N*, %)			
Emergency department	18 (69.2)	25 (62.5)	47 (63.5)
Inpatient	8 (30.8)	15 (37.5)	27 (36.5)
Nondisclosure visit (*N*, %)			
Emergency department	N/A	26 (65)	49 (67.1)
Inpatient	N/A	14 (35)	24 (32.9)
* McNemar’s χ2*	*N/A*	*0.14 (p* = *.71)*	*0.47 (p* = *.49)*
Presenting complaint			
Disclosure visit (*N*, %)			
Medical	7 (26.9)	9 (22.5)	46 (62.2)
Psychosocial	19 (73.1)	31 (77.5)	25 (37.8)
Nondisclosure visit (*N*, %)			
Medical	N/A	9 (22.5)	69 (93.2)
Psychosocial	N/A	31 (77.5)	5 (6.8)
* McNemar’s χ2*	*N/A*	*0 (1)*	*18.4 (p* < *.001)*
Evaluator at disclosure visit (*N*, %)			
Psychologist, psychiatrist or social worker	26 (100)	35 (87.5)	33 (44.6)
ED physician	0 (0)	1 (2.5)	6 (8.1)
No evaluation conducted	0 (0)	4 (10)	35 (47.3)
Disposition at disclosure visit (*N*, %)			
Inpatient care	2 (7.7)	8 (20)	4 (5.4)
Partial hospital or intensive outpatient program	11 (42.3)	18 (45)	19 (25.7)
Outpatient/residential	12 (46.5)	11 (27.5)	17 (23)
No recommendations	1 (3.9)	3 (7.5)	34 (46)
Current Suicidal Ideation (ASQ question one, two, or three positive)			
Disclosure visit (*N*, %)	22 (81.5)	33 (82.5)	38 (51.3)
Nondisclosure visit (*N*, %)	N/A	12 (30)	5 (6.8)
* McNemar’s χ2*	*N/A*	*3.7 (p* = *.06)*	*65.1 (p* < *.001)*
Imminent Risk (ASQ question five positive)			
Disclosure visit (*N*, %)	2 (7.7)	3 (7.5)	4 (5.4)
Nondisclosure visit (*N*, %)	N/A	3 (7.5)	0 (0)
* McNemar’s χ2*	*N/A*	*0 (p* = *1)*	*4 (p* = *.05)*

*ASQ* Ask Suicide Questions, *N/A* not applicable

Among the 40 visit pairs where the patient received a full psychiatric assessment at the nondisclosure visit, 35 (87.5%) of patients were evaluated by a psychiatric consultant at the disclosure visit, 1 (2.5%) was evaluated by an ED physician, and 4 (10%) were missed (not evaluated) at the disclosure visit. There was no significant difference between ED and inpatient settings. These visit pairs tended to have psychiatric or social presenting complaints at both the disclosure (77.5% psychosocial) and nondisclosure (77.5% psychosocial) visits. Positive responses on ASQ questions 1, 2, or 3, indicating recent suicidal ideation, occurred in 82.5% of disclosure visits and 30% of nondisclosure visits (*p* = 0.06); while few (7.5%) of both the disclosure and nondisclosure visits included imminent risk (positive response to ASQ question five). Patients received recommendations for follow-up psychiatric care at 92.5% of disclosure visits in this sample.

Among the 74 visit pairs in which no evaluation of the previously disclosed attempt occurred at the nondisclosure visit, the disclosure history was originally evaluated by a psychiatric consultant in 33 (44.6%) visits, by an ED physician in 6 (8.1%) visits, and missed (not evaluated) in 35 (47.3%) visits. There was no significant difference between ED and inpatient settings. The presenting complaint tended to be medical rather than psychiatric or social in both the disclosure visits (62.2% medical) and the nondisclosure visits (93.2% medical); the proportion of medical presenting complaints was significantly greater in the nondisclosure than disclosure visits (*p* < 0.001). ASQ questions one, two, or three were more likely to be positive in the disclosure visits (51.3%) than in nondisclosure visits (6.8%, *p* < 0.001). ASQ question five (imminent risk of suicide) was positive in 4 (5.4%) of the disclosure visits. Psychiatric follow-up recommendations for the disclosure visits included inpatient care (5.4%), partial hospitalization or intensive outpatient care (25.7%), outpatient care or return to a residential facility (23%), or no recommendation (46%). No recommendations for psychiatric follow-up occurred at the nondisclosure visits. Of these 74 visit pairs, nearly half (47.3%) of the initial disclosures of past suicide attempts were missed, meaning they were followed by no further assessment.

## Discussion

This study aimed to investigate the phenomenon of denial of a historic suicide attempt following previous disclosure of an attempt in the context of universal suicide screening in a pediatric hospital. In this analysis of over 23,000 inpatient and ED encounters, we identified 74 clinically significant instances in which patients who had disclosed a history of suicide attempt denied that history in a subsequent visit. Nondisclosure was significantly associated with medical rather than psychosocial presenting complaints. Compared with disclosure visits, nondisclosure was also associated with denial of recent suicidal ideation captured by other items on the ASQ screener. Nondisclosure of a history of suicide attempts may represent missed opportunities for further assessment of high-risk individuals.

The rate of positive ASQ screenings in our sample was 14.7% (*N* = 3433). While the majority of positive screenings in our sample (85.2%) were from encounters for a medical presenting concern, the odds of having a positive screener were higher for psychiatric versus medical complaints. Our ASQ positivity rates are similar to findings from Roaten et al. (2021) who found a higher proportion of positive screens for encounters involving a psychiatric presenting complaint. However, their sample had a lower rate of overall positivity (27.6% for psychiatric presenting complaints, 2.3% for medical complaints), likely due to their inclusion of outpatient in addition to inpatient and ED data. Demographic factors associated with positive screens were similar in our sample to those of Roaten et al. (2021), including increased risk among psychiatric versus nonpsychiatric encounters, as well as adolescent encounters being more likely to result in a positive screening compared with pre-teens. Overall, 8.5% of unique patients screened endorsed a lifetime suicide attempt, consistent with data from the 2019 Youth Risk Behavior Survey in which 8.9% of youth reported a lifetime suicide attempt (Gaylor et al., 2023).

Of the patients who endorsed a history of suicidal behavior in at least 1 screening (*N* = 1460), 8.7% individuals subsequently denied a historic attempt. Our findings suggest that question four on the ASQ has overall good test–retest reliability, but nondisclosure is not uncommon. We found that nondisclosure of a previously disclosed suicide attempt may be related to the initial disclosure visit, to the nondisclosure visit, or other aspects of the individual’s treatment in the between-visit interval. Outcomes at the disclosure visit included full evaluations and dispositions to psychiatric follow-up for some patients and no evaluation for others. Notably, 47% of youth whose nondisclosure was classified as clinically significant were not properly assessed during the disclosure visit following positive screening. Such issues with “misses” could adversely impact youth’s willingness to re-disclose, as disclosure had not previously resulted in a response from providers. As nondisclosure occurred more often during encounters for a medical presenting concern, it may be that the patient preferred to avoid further psychiatric assessment and so denied a history of suicide attempt when asked. Nondisclosure was also significantly negatively associated with recent suicidal ideation as only 6.8% of nondisclosure visits had a positive response on ASQ questions one, two, or three. While details of psychiatric care between nondisclosure and disclosure visits are beyond the scope of this paper, chart review indicates that some had completed intensive psychiatric treatment in the between-visit interval. However, it is also possible that youth who deny a previously disclosed attempt similarly deny suicidal ideation even if it is present. Qualitative research is warranted to determine whether this association is driven by a desire to deny all suicidal ideation and behavior due to perceptions that disclosure will lead to hospitalization. Further, analyses investigating additional disclosure visit factors such as length of visit might illuminate additional drivers of nondisclosure. There is currently a dearth of literature on the clinical impact of conflicting screenings of question four of the ASQ, and in the context of universal screening, repeated administration will provide ample opportunities for nondisclosure. It is vital that researchers explore the drivers of clinically significant nondisclosure to inform the broad implementation of universal screening. The frequency of subsequent non-disclosure suggests that it will be important for clinicians to have a mechanism to review historical screeners in the EMR to determine whether further assessment is indicated. It would be beneficial for researchers to involve youth with lived experience to develop guidance regarding how to proceed when historic attempts are denied.

Despite the well-established psychometric properties of the ASQ, there is limited information regarding how youth respond to being asked about a history of suicidal behavior when administered repeatedly. Our results indicate that youth who choose not to disclose a previously disclosed suicide attempt upon repeated assessment are likely to present to their nondisclosure visit with a medical complaint and without endorsing recent suicidal ideation. Nondisclosure often occurred when positive screens were not followed by assessment and safety planning. These findings lay the groundwork for qualitative work with youth with lived experience with disclosure and subsequent denial of prior attempts to better understand the variables that contribute to such nondisclosure.

In reviewing the electronic medical record, it is unclear as to whether providers employed the four supplemental screening questions for patients who only screen positive only due to a historic attempt, which constitutes a limitation of the current study. Some of our participants who were classified as clinically significant nondisclosures may have answered those follow-up questions in a way that allowed them to exit the pathway. This lack of fidelity data constitutes a limitation to the current study. It is important to build toolkits in the EMR which reflect this completion of the screener with follow-up questions when patients identify a historic attempt alone. One next step for our setting will be to make changes to the toolkit to reflect this. The current study used EMR data to complete a retrospective chart review to capitalize on existing data; this value of this methodology is limited by variance in the completion of documentation. EMR documentation can vary in precision and does not allow us to draw conclusions about cause and effect. Despite limitations, this study highlighted several factors associated with subsequent nondisclosure of a suicide attempt.

In conclusion, our study revealed that youth who choose not to disclose a previously disclosed suicide attempt upon repeated assessment are more likely to present with a medical concern than a psychiatric concern to their nondisclosure visit. Nondisclosure also often occurs when initial disclosure screeners are not followed by assessment and safety planning. Our findings illuminate the need for future studies aimed at better understanding the reasons why youth may not disclose a previously disclosed suicide attempt and lay the groundwork for qualitative work with youth with lived experience.

## Data Availability

Data are not available due to privacy concerns due to the nature of the qualitative analyses.
